# The role of mechanobiology on the Schwann cell response: A tissue engineering perspective

**DOI:** 10.3389/fncel.2022.948454

**Published:** 2022-08-10

**Authors:** Phanee Manganas, Paraskevi Kavatzikidou, Antonis Kordas, Eleftheria Babaliari, Emmanuel Stratakis, Anthi Ranella

**Affiliations:** ^1^Tissue Engineering, Regenerative Medicine and Immunoengineering Laboratory, Institute of Electronic Structure and Laser, Foundation for Research and Technology-Hellas (IESL-FORTH), Heraklion, Greece; ^2^Ultrafast Laser Micro and Nano Processing Laboratory, Institute of Electronic Structure and Laser, Foundation for Research and Technology-Hellas (IESL-FORTH), Heraklion, Greece; ^3^Department of Materials Science and Technology, University of Crete, Heraklion, Greece

**Keywords:** mechanobiology, Schwann cells, neural tissue engineering, mechanical properties, topography, shear stress

## Abstract

Schwann cells (SCs), the glial cells of the peripheral nervous system (PNS), do not only form myelin sheaths thereby insulating the electrical signal propagated by the axons, but also play an essential role in the regeneration of injured axons. SCs are inextricably connected with their extracellular environment and the mechanical stimuli that are received determine their response during development, myelination and injuries. To this end, the mechanobiological response of SCs is being actively researched, as it can determine the suitability of fabricated scaffolds for tissue engineering and regenerative medicine applications. There is growing evidence that SCs are sensitive to changes in the mechanical properties of the surrounding environment (such as the type of material, its elasticity and stiffness), different topographical features provided by the environment, as well as shear stress. In this review, we explore how different mechanical stimuli affect SC behaviour and highlight the importance of exploring many different avenues when designing scaffolds for the repair of PNS injuries.

## Introduction

The nervous system of vertebrates consists of the central nervous system (CNS) and the peripheral nervous system (PNS). Both are made up of neurons and glial cells, with neurons being responsible for receiving and transmitting electrical and chemical signals and glial cells providing the necessary support and protection for neurons. In the PNS, the glial cells are known as Schwann cells (SCs) and are responsible for the creation of myelin sheaths that protect and insulate the axons of peripheral neurons (Belin et al., 2017). They are also essential and indispensable for axon regeneration in the event of injury (Rosso et al., 2017) and as such, their role and function are under much investigation.

One of the main differences between the CNS and PNS is their capacity for regeneration. Injuries in the PNS are more easily repaired, as broken myelin sheaths can be phagocytosed and removed from the area of injury (Yao and Priyadarshani, 2018), while in the CNS, there are a number of limiting factors—including glial scar formation and the presence of inhibitory molecules—that make regeneration next to impossible (Huebner and Strittmatter, 2009). In the case of the PNS, when an injury is quite small, it can be repaired by suturing the two ends of the severed nerve together. Autografts have been considered the standard method of countering PNS injuries with slightly larger gap lengths, while commercially available nerve guides have been successful for nerve gaps of 20 mm or more (Bell and Haycock, 2012). However, despite the fact that there are treatments available for PNS injuries, there are still factors that limit their application, such as donor nerve shortage or immunological issues, and they have displayed moderate success (Millesi and Tsolakidis, 2005; Vindigni et al., 2009; Mahumane et al., 2018). As a result, there is a constant ongoing effort to find alternative treatment options by utilising the principles of tissue engineering.

Neural tissue engineering takes advantage of a large number of different biofabrication techniques, as well as biomaterials in order to create three-dimensional scaffolds and structures that can be used to facilitate regeneration (Boni et al., 2018; Papadimitriou et al., 2020; Doblado et al., 2021; Scaccini et al., 2021). The aim when creating scaffolds for such purposes is to mimic the physiological environment as closely as possible and provide the necessary cues to promote repair while ensuring that no localised toxicity or immune reaction is induced (Crupi et al., 2015; Doblado et al., 2021). It is well known that SCs are inextricably connected to their extracellular environment (Rosso et al., 2017). Characteristics of their physiological microenvironment include—but are not limited to—specific mechanical properties (such as elasticity and stiffness), different topographical features, as well as shear stress. All these factors are taken into account when designing scaffolds targeting PNS injuries, either individually or collectively.

Mechanobiology is related to how cells, tissues and organs sense the surrounding mechanical and physical signals and how are these signals converted into specific cellular responses such as adhesion, spreading, migration, gene expression, and cell-cell interactions in multiple cell types (Jansen et al., 2015; Belin et al., 2017). Mechanobiology relies on two main players within the cell: (i) mechanosensors, that allow cells to sense the mechanical signals provided by their environment; and; (ii) mechanotransducers, which enable cells to convert mechanical cues into biochemical signals. Various mechanosensors and mechanotransducers from both intra- and extracellular compartments have already been identified (Jansen et al., 2015). In the case of peripheral nerves and SCs, their identities are still emerging, with research focusing on how physical signals can be transmitted in SCs through the extracellular matrix (ECM), cell adhesion molecules (CAMs) and internal structures, such as the cortical cytoskeleton and the nucleus.

In this review, we explore how the different extracellular stimuli affect SC behaviour and highlight the importance of exploring many different avenues when designing scaffolds for the repair of PNS injuries.

## The Effect of The Microenvironment on Schwann Cell Behaviour

### The effect of mechanical properties

Schwann cells in peripheral nerves are physiologically exposed to mechanical stimuli such as shear and compressive and tensile stress, which can occur due to injuries or diseases, as well as during development and adulthood (Zhang et al., 2015; Belin et al., 2017). Peripheral nerves possess great elasticity in order to propagate action potentials throughout developmental growth (Simpson et al., 2013), mechanical compression and stretches while performing daily activities (Phillips et al., 2004). SCs are very sensitive to the surrounding stiffness and possess great plasticity. In case of injury of the peripheral nerves, myelinated SCs can dedifferentiate and guide the regeneration of peripheral axons (Jessen and Mirsky, 2016; Boerboom et al., 2017). Generally, in peripheral nerves, myelinated fibres are surrounded by 6–15 layers of connective tissue, which shield the SCs and the axons from mechanical forces originating from the external environment. It is known that the relative elasticity or stiffness of the peripheral nerves can affect the mechanical cues that the SCs are exposed to, while SC architecture and basal lamina integrity play key roles in the SC response to mechanical stress (Belin et al., 2017). *In vitro* studies have shown that mechanical stimulation at low levels may activate SC mitogenic pathways, independently of the regulation occurring between SCs and axons after axonal injury (Salzer and Bunge, 1980).

The mechanical properties of the ECM are defined mainly by elastin and collagen fibres that provide resilience (elasticity) and tensile strength. In peripheral nerves, there are more collagen fibres in comparison to elastin fibres (Sunderland, 1965), which contribute to the elastic properties of peripheral nerves. The main mechanotransducers that have been identified in SCs are responsible for the transmission of signals through the ECM and the SC basal side. Various studies report that SCs interact with axons on their apical side, where the biochemical signals from neurons are critical for SC migration, proliferation, survival, polarisation, differentiation, and gene expression (Monk et al., 2015; Salzer, 2015). SC function depends on the formation of adhesion complexes (through CAMs) between SCs with both axons and the ECM. All these stimuli are then transduced into biological responses, by YAP/TAZ, MRTF or LINC (Poitelon et al., 2016). In addition, forces such as mechanical compression can act on the actin cytoskeleton, leading to deformation of the nucleus and influencing chromatin organisation (Hernandez et al., 2016), while the nuclear envelope and the actin cytoskeleton work as mechanotransducers between the inner membrane of the cell and the nucleus (Plessner et al., 2015). Rosso et al. (2022) showed that the intrinsic physiological plasticity of SCs, which change their phenotype in response to physiological and patho-physiological changes in their microenvironment, in conjunction with their demonstrated mechanosensitivity, render them powerful targets for cell-based regenerative therapies.

In the field of peripheral nerve engineering, there is a wide range of biomaterials, both natural and synthetic, applied to the supporting cells involved in the repair process, such as neurons, SCs, macrophages, and blood vessels (Gregory and Phillips, 2021; Powell et al., 2021). Synthetic polymers are popular as their mechanical properties can be fine-tuned and they can be adapted to improve cell adhesion (Shahriari et al., 2017). Natural materials (typically derived from ECM components and found in hydrogel form) give the benefit of structural integrity for supporting regenerating axons, whilst maintaining the materials’ viscoelastic properties and their capability to further improve function (Bhatnagar et al., 2016). They can also be adapted to possess additional functionalities, such as controlled drug release to *in situ* gel formation. They can also be easily adjusted to fit defects with complicated geometries, such as that of the spinal cord. Multiple biomaterials such as chitosan (Li et al., 2014), collagen (Dalamagkas et al., 2016; Yeh et al., 2020), hyaluronic acid (Thomas et al., 2017), polycaprolactone (PCL; Chew et al., 2008; Mobasseri et al., 2014), poly (lactic acid; PLA; Miller et al., 2001; Mobasseri et al., 2014), poly (lactide-co-glycolide; PLGA; Babaliari et al., 2018a), and others (Gu et al., 2011; Bell and Haycock, 2012; Lotfi et al., 2019) have been assessed for scaffold fabrication. One of the most common materials used for hybrid polymeric conduits is gelatin/polycaprolactone (PCL), which has been shown to provide great support for *in vitro* neurite outgrowth and SC proliferation (Boni et al., 2018).

The surrounding ECM and microenvironment play a significant role in peripheral nerve tissue regeneration. Studies have shown that the regularity of a scaffold surface can guide migration and promote the maturation of SCs, and consequently direct the growth of dorsal root ganglion (DRG) neuritis (Ning et al., 2018; Petcu et al., 2018). In another study, Chen and co-workers evaluated the effects of cyclic tensile stimulation on the neural differentiation capabilities of human SCs. The auxetic hydrogels were found to withstand up to 20% tensile strain without tears, while only losing about 10% weight after being immersed for 14 days. The tensile forces were able to enhance cell viability and proliferation compared to static culture (Chen et al., 2020). Mechanical stimuli can also affect the remyelination of injured axons, with factors such as surface area, porosity, and surface structuring playing an important role in supporting and regulating SCs and directly influencing myelin-related gene expression (Liu et al., 2019; Park et al., 2021).

From the above, it can be seen that when it comes to assessing scaffold requirements for peripheral nerve tissue engineering, one must always take into account: (a) the static mechanical properties of the scaffolds whose aim is to imitate nerve tissue stiffness; (b) the dynamic nature of the nerve tissue; and (c) the time-varying mechanics relating to the stress gradient responses in the native tissue.

### The effect of topography

The effect of topography —the surface spatial features of tissues or biomaterials—on SCs has attracted a lot of attention in the fields of neuron regeneration and regenerative medicine (Lotfi et al., 2019). Topographical cues in the micro-and nano- scale affect key SC responses such as migration, alignment, proliferation, and differentiation. The importance of these effects is highlighted by the fact that SCs provide structural support, remove debris, and direct axon regrowth in diseases associated with the PNS. Researchers attempt to mimic the natural environment of SCs to provide all the necessary cues that will enhance SC performance when developing strategies to counter neurodegenerative diseases, trauma, and disorders (Gu et al., 2011; Bell and Haycock, 2012; Lotfi et al., 2019). Fabrication of suitable scaffolds for SC seeding is gaining more popularity as a means to develop grafts that can be used as transplants for SC culture and neuron regeneration for *in vivo* trials (Gu et al., 2011; Deng et al., 2021).

Various topographical features have been investigated in literature (Lotfi et al., 2019). These features include but are not limited to, micro- and nano-scale surface topography and patterning (Miller et al., 2001; Schmalenberg and Uhrich, 2005; Li et al., 2014; Yiannakou et al., 2017; Angelaki et al., 2020), cell imprinted topography (Moosazadeh Moghaddam et al., 2019), scaffold geometry (grooves, filaments, wells, pillars etc.; Schmalenberg and Uhrich, 2005; Chew et al., 2008; Mitchel and Hoffman-Kim, 2011; Mobasseri et al., 2014; Tonazzini et al., 2015; Chen et al., 2019), hydrophilicity (Mobasseri et al., 2014), and roughness (Simitzi et al., 2015; Babaliari et al., 2021; Huang et al., 2021) and porosity (Li et al., 2014). Moreover, topographical characteristics are not investigated in a qualitative manner exclusively. For instance, grooves of different dimensions and spacing have been reported to have different effects on SCs (Schmalenberg and Uhrich, 2005; Li et al., 2014; Tonazzini et al., 2015). It has been shown that surface coating further mimics the ECM, which in turn boosts SC performance in axon regeneration. In 2001, Miller et al. (2001) seeded SCs and DRGs on laminin-coated poly(D, L-lactic acid) micropatterned grooves with varied groove spacing and depth and reported the effects of topography alongside the benefit of SC presence as a biological cue for neurite outgrowth and alignment. Another study reports the use of laminin-coated poly (dimethylsiloxane; PDMS) grooves for SC alignment as a means of directing neuron regeneration (Schmalenberg and Uhrich, 2005). Mobasseri et al. (2014) developed PCL/PLA films as scaffolds and reported the combined effect of different groove geometries and shapes along with scaffold wettability on SC interactions. Ahmed and Brown have also reported differential SC responses such as adhesion, alignment and migration on fibronectin fibres by comparing different combinations of substrates of fibronectin, laminin, and poly (L-lysine) and highlighting the directed orientation and enhanced speed, cell extension and cell area SCs exhibited on fibronectin fibres compared to the respective responses on non-topographical substrates (Ahmed and Brown, 1999).

Although the importance of topography cannot be understated, it is more common to investigate the topographical effects alongside other stimuli, such as electrical conductivity, shear stress, and use of growth factors, and examine their synergistic or antagonistic effects on SC behaviour. For instance, Huang et al. (2021) have developed reduced graphene oxide electrospun fibres that exhibit electrical conductivity and are suitable for SC electrical stimulation (ES). In another study, SCs combined with glial-derived neurotrophic factor (GDNF) on laminin-coated filaments exhibited significant and unidirectional axonal growth in the graft environment, reducing the inflammatory response, astrogliosis and tissue damage compared to non-coated filaments or coated filaments lacking SCs/GDNF. This demonstrated that the combination of laminin-coating and the use of SCs/GDNF was key to the success of the approach (Deng et al., 2021).

Another point of interest pertains to studies that have focused on co-culturing neurons alongside SCs since the nervous system is very complicated and multiple parameters should be taken into account for the successful application of scaffolds as *in vivo* transplants (Angelaki et al., 2020; Kordas et al., 2022). One such study utilised a substrate with combined morphology including both micro-cones and nano-ripples for the co-culture of SCs and neuronal cells. The authors were able to show that SCs adhesion was affected by the underlying pattern, which in turn also influenced neuronal cell behaviour (Angelaki et al., 2020).

Additionally, the focus has been directed not only on morphology, directionality and axon regeneration, but also on other key responses such as remyelination, myelin-related gene, and neurotrophic factor regulation, and examination of markers of immature or aged SCs. For example, Chew et al. (2008) used electrospun fibrous scaffolds to study SC alignment and investigate the regulation of various selected genes, myelin-specific proteins and immature SC markers. Follow-up work also highlighted the importance of topography on the spatial organisation of SCs, which in turn influenced myelination as well as neurite alignment (Siddiqui et al., 2021). Another study also used electrospun scaffolds and investigated topographical effects on myelin-related genes such as myelin-associated glycoprotein (MAG) and myelin protein zero (P0) *via* qRT-PCR, enabling the creation of a gene expression profile during the myelination process on scaffolds with specific alignment (Radhakrishnan et al., 2015).

It is evident that topography is one of the major factors that influence SC behaviour and plays a pivotal role in nerve regeneration applications. Topography can influence major cell responses such as adhesion, migration, alignment, and proliferation, while also affecting the regulation of multiple genes related to myelination and neurodegenerative diseases. As such, it is essential that it is taken into account and integrated into potential solutions.

### The effect of shear stress

When studying cell-material interactions, more often than not conventional cell culture techniques are used, which means that the cells are cultured in flasks, Petri dishes, and other surfaces under static conditions (Coluccio et al., 2019), where they also have limited cell-cell interactions. However, within a multicellular organism, cells interact with a number of materials with different mechanical properties and topographical characteristics and are surrounded by fluid and nutrients (Zhang and Van Noort, 2011; Babaliari et al., 2018b). In order to better understand biological problems, it is important to assess cellular behaviour under conditions that reflect more closely the *in vivo* conditions with cell-cell, cell-matrix, and cell-soluble factor interactions (Hui and Bhatia, 2007). To achieve a more realistic environment for biological research, microfluidic devices are routinely used, as they can offer precise control over the microenvironment that influences biochemical and mechanical factors in a cell and, thus, cell functionality, such as changes in the flow-induced shear stress, the pH or O_2_ levels (Zhang and Van Noort, 2011).

Although shear stress—the external force acting on an object or surface parallel to the slope or plane in which it lies—is a critical component of the natural environment for the regeneration of axons (Chafik et al., 2003), the use of such systems for the study of neuronal cell behaviour is not widespread and there are very few studies available. One such study investigated the use of different substrate coatings for the use of SCs in microfluidic culturing environments, as shear stress significantly affected the effectiveness of surface coatings (Chafik et al., 2003). Gupta and co-workers studied the effect of shear stress on SC proliferation and genetic expression profiles and were able to find that under constant laminar fluid flow SCs had increased proliferation rates but displayed downregulation of MAG and myelin basic protein (MBP; Gupta and Steward, 2003; Gupta et al., 2005). Babaliari et al. (2021) have studied the combined effect of shear stress and topography on SC behaviour under dynamic culture conditions attained *via* continuous flow. Experiments using a precise flow-controlled microfluidic system which incorporated laser-microstructured microgrooved polyethylene terephthalate (PET) substrates revealed that, depending on the relation of the direction of the flow with respect to the topographical features (parallel or perpendicular), wall shear stress gradients act in a synergistic or antagonistic manner to topography in promoting a guided morphological cell response (Babaliari et al., 2021).

It is thus evident that flow-induced shear stress also affects neuronal behaviour. As a result, the necessity of developing *in vitro* biomimetic cell culture systems simulating shear stress, together with the micro/nano topography of the *in vivo* environment is mandatory. By utilising such systems to study the mechanobiology of peripheral glial cells, there would be a great benefit for various applications, including the creation of autologous graft substitutes for nerve tissue regeneration.

## Conclusion and Future Perspectives

**Figure 1:**
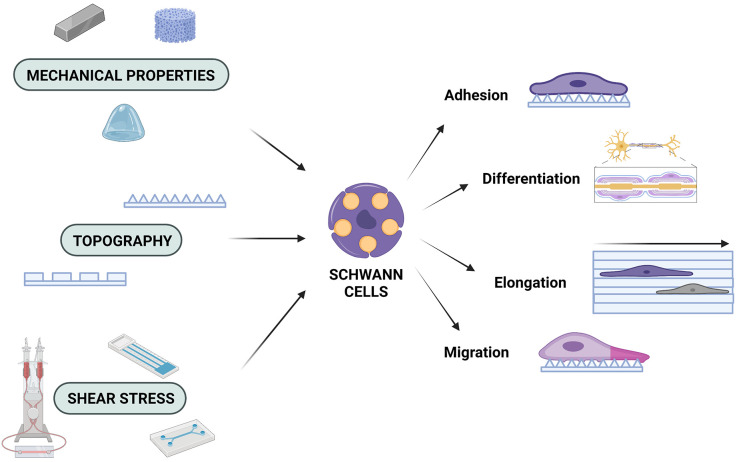
Mechanobiology and the Schwann cell response. SCs are affected by the mechanical properties of the engineered microenvironment, the underlying topography, and shear stress forces, which can determine their adhesion profiles, their ability to migrate and/or elongate, as well as their capacity to differentiate. Created with https://biorender.com.

Over the past decades, there has been great interest in trying to understand the function of SC cells in order to fully utilise their potential in tissue engineering. As with all cells, SC function is inextricably connected to the microenvironment and it must not be ignored when attempting to understand PNS injury and disease. In this review, it has been highlighted how different aspects of the microenvironment (mechanical properties, topography and shear stress) can influence SC behaviour (Figure 1). It must be noted that all of these properties are so interconnected that integral parts of the process can be missed when they are not all taken into account. Due to the complexity of the nervous system, it is more beneficial to assess and investigate all cues provided by the natural microenvironment and apply this knowledge to the fabrication of biomimetic scaffolds. By integrating different mechanobiological aspects into scaffold design, potential synergistic, or antagonistic effects that could affect scaffold performance can be investigated. In addition, by using a multifaceted approach in scaffold design, the immunogenicity can also be modulated. By selecting materials with appropriate chemical and mechanical characteristics, as well as carefully designing the 3D architecture of the scaffolds, the immune response could be controlled and thus, lead to the reduction of adverse effects, such as scar formation (Crupi et al., 2015; Andorko and Jewell, 2017). Scaffolds that combine such features are gaining more ground as candidate conduits for *in vivo* PNS regeneration (Bell and Haycock, 2012; Liu et al., 2021). Hence, by choosing the appropriate materials, as well as topographical, mechanical, and chemical properties for potential scaffolds, while also utilising stimuli such as electrical stimulation and shear stress, more comprehensive solutions can be offered, which will pave the way for future state-of-the-art scaffolds to counter injuries and neurodegenerative diseases (Tupone et al., 2021).

## Author Contributions

PM, PK, AK, and EB wrote the manuscript. PM created the figure. ES revised the manuscript. AR designed and revised the manuscript. All authors contributed to the article and approved the submitted version.

## Funding

This work was funded by the projects “BioCombs4Nanofibers” (Horizon 2020 FET Open Programme—GA862016), “NeuroStimSpinal” (Horizon 2020 Research and Innovation Programme—GA829060), and by the Stavros Niarchos Foundation within the framework of the project ARCHERS (“Advancing Young Researchers’ Human Capital in Cutting Edge Technologies in the Preservation of Cultural Heritage and the Tackling of Societal Challenges”; Reference Number 2019_1193).

## Conflict of Interest

The authors declare that the research was conducted in the absence of any commercial or financial relationships that could be construed as a potential conflict of interest.

## Publisher’s Note

All claims expressed in this article are solely those of the authors and do not necessarily represent those of their affiliated organizations, or those of the publisher, the editors and the reviewers. Any product that may be evaluated in this article, or claim that may be made by its manufacturer, is not guaranteed or endorsed by the publisher.
